# Effects of a Home‐Based Physical Rehabilitation Program on Tibial Bone Structure, Density, and Strength After Hip Fracture: A Secondary Analysis of a Randomized Controlled Trial

**DOI:** 10.1002/jbm4.10175

**Published:** 2019-03-06

**Authors:** Tuuli H Suominen, Johanna Edgren, Anu Salpakoski, Marja Arkela, Mauri Kallinen, Tomas Cervinka, Timo Rantalainen, Timo Törmäkangas, Ari Heinonen, Sarianna Sipilä

**Affiliations:** ^1^ Gerontology Research Center Faculty of Sport and Health Sciences University of Jyväskylä Jyväskylä Finland; ^2^ Rehabilitation Center Peurunka Laukaa Finland; ^3^ Department of Physical and Rehabilitation Medicine Central Finland Central Hospital Jyväskylä Finland; ^4^ Department of Medical Rehabilitation Oulu University Hospital and Center for Life Course Health Research University of Oulu Oulu Finland; ^5^ Satakunta University of Applied Sciences Pori Finland; ^6^ Faculty of Sport and Health Sciences University of Jyväskylä Jyväskylä Finland

**Keywords:** AGING, BONE QCT/μCT, CLINICAL TRIALS, EXERCISE, INJURY/FRACTURE HEALING

## Abstract

Weight‐bearing physical activity may decrease or prevent bone deterioration after hip fracture. This study investigated the effects of a home‐based physical rehabilitation program on tibial bone traits in older hip fracture patients. A population‐based clinical sample of men and women operated for hip fracture (mean age 80 years, 78% women) was randomly assigned into an intervention (*n *= 40) and a standard care control group (*n *= 41) on average 10 weeks postfracture. The intervention group participated in a 12‐month home‐based rehabilitation intervention, including evaluation and modification of environmental hazards, guidance for safe walking, nonpharmacological pain management, motivational physical activity counseling, and a progressive, weight‐bearing home exercise program comprising strengthening exercises for the lower legs, balance training, functional exercises, and stretching. All participants received standard care. Distal tibia (5% proximal to the distal end plate) compressive bone strength index (BSI; g^2^/cm^4^), total volumetric BMD (vBMD_TOT_; mg/cm^3^), and total area (CSA_TOT_; mm^2^), as well as midtibia (55%) strength–strain index (SSI; mm^3^), cortical vBMD (vBMD_CO_; mg/cm^3^), and ratio of cortical to total area (CSA_CO_/CSA_TOT)_ were assessed in both legs by pQCT at baseline and at 3, 6, and 12 months. The intervention had no effect (group × time) on either the distal or midtibial bone traits. At the distal site, BSI of both legs, vBMD_TOT_ of the fractured side, and CSA_TOT_ of the nonfractured side decreased significantly over time in both groups 0.7% to 3.1% (12 months, *p* < 0.05). At the midshaft site, CSA_CO_/CSA_TOT_ and SSI of both legs, and vBMD_CO_ of the fractured leg, decreased significantly over time in both groups 1.1% to 1.9% (12 months, *p* < 0.05). Trabecular and cortical bone traits of the tibia on the fractured and the nonfractured side deteriorated throughout follow‐up. The home‐based physical rehabilitation intervention aimed at promoting mobility recovery was unable to prevent bone deterioration in older people after hip fracture. © 2019 The Authors. *JBMR Plus* published by Wiley Periodicals, Inc. on behalf of American Society for Bone and Mineral Research.

## Introduction

The substantial and long‐term decline in bone properties that occurs after hip fracture^1^, ^2^, ^3^, ^4^, ^5^ markedly increases the risk for a second fracture.^6^, ^7^ In the contralateral hip, as measured by DXA, the loss of bone density, structure, and strength over the year after fracture far exceeds the decrements from normal aging, in both men and women.^1^, ^3^, ^4^, ^5^ Cross‐sectional studies using peripheral 3D‐imaging modalities have also revealed marked impairments in tibial properties on both the fractured and nonfractured sides.^2^, ^8^ These reductions were most evident in bone geometric properties^2^, ^8^ and correlated with hip BMD measured by DXA.^8^ In our previous study,^2^ with individuals on average 3.5 years post hip fracture, a considerable and persistent side‐to‐side difference in geometric properties favoring the nonfractured leg was observed. Part of this bone loss was presumably caused by disuse of the affected limb.

Bone‐loading physical activity may decrease or prevent the postfracture deterioration of bone properties. As summarized in meta‐analyses and reviews,^9^, ^10^, ^11^ the most effective physical activity programs for increasing or preserving bone health in older populations incorporate progressive resistance and power training, weight‐bearing impact loading activities, or challenging balance and agility training. Most of the previous studies have, however, focused on relatively healthy populations, whereas only a few studies have been performed in the frail elderly,^12^, ^13^, ^14^ and even fewer in hip fracture patients.^15^, ^16^ Furthermore, the findings from the limited number of trials examining the effects of exercise on bone structure, strength, and volumetric density (vBMD) in older people are conflicting^17^, ^18^, ^19^, ^20^, ^21^, ^22^ and no studies involving 3D bone characterization have been conducted in hip fracture patients or subjects comparable to them. Thus, it is currently unclear whether fragile bones, such as those in older hip fracture patients, are able to adapt to increased loading.

To date, no attempts have been made to investigate the effects of physical exercise on bone structural and densitometric traits of both legs after hip fracture. Although exercise has increased muscle strength and functional capacity in older people with a recent hip fracture,^15^, ^23^, ^24^ the osteogenic effects remain unclear. We hypothesized that a 12‐month home‐based physical rehabilitation program, including weight‐bearing exercises, would be feasible and effective in reducing postfractural losses in tibial bone density, structure, and strength in older people recovering from a recent hip fracture.

## Subjects and Methods

### Design and participants

**Figure 1:**
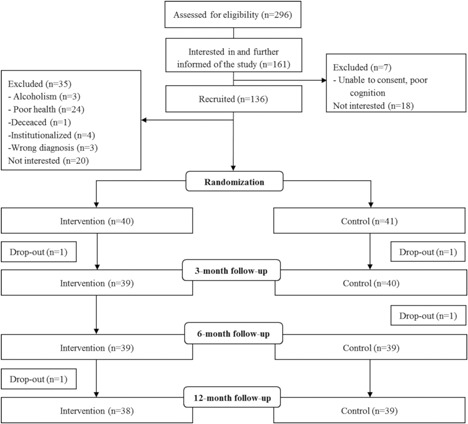
Flowchart of the study.

This study was a 12‐month randomized controlled trial (RCT; ISRCTN53680197; Fig. 1) investigating the effects of a home‐based rehabilitation program on mobility recovery among community‐dwelling older people with a recent hip fracture.^25^ This secondary analysis reports the effects of the intervention on tibial bone traits. The design and recruitment procedure have been published in detail before.^26^ Briefly, patient records at the Central Finland Central Hospital (Jyväskylä, Finland) were reviewed to recruit all ambulatory and community‐dwelling men and women over age 60 years who had been operated for a femoral neck or pretrochanteric fracture (ICD code S72.0 or S72.1) between 1.3.2008 and 31.12.2010, and were resident in the catchment area. In total, 269 men and women were informed about the study. Of these, 161 expressed interest in the study and were visited by a researcher during their inpatient stay at the health care center for a preliminary assessment of eligibility. Thereafter, 136 persons were invited to the baseline measurements, of whom 81 eligible patients participated in the study. The exclusion criteria were severe memory problems (Mini Mental State Examination <18), alcoholism, a severe cardiovascular or pulmonary condition or some other progressive disease, and severe depression (Beck Depression Inventory >29).

After the baseline measurements, conducted on average 10 weeks postfracture, the participants were randomized into an intervention (*n* = 40) and a standard care control (*n* = 41) group using a computer‐generated group allocation list generated by a blinded statistician, who was not involved in either the recruitment or data collection processes. Blocks of 10, stratified by gender and surgical procedure (internal fixation versus arthroplasty), were used.

Follow‐up measurements were arranged at 3, 6, and 12 months after the baseline measurements. All assessments were conducted at the research laboratory. All outcome assessors were blinded to the treatment‐group assignment. All participants gave their written informed consent and permission to review their medical records prior to participation in the study. The study was approved by the Ethics Committee of the Central Finland Health Care District (Dnro56/2007) and conformed to the principles of the Declaration of Helsinki.

### Peripheral quantitative computed tomography

Properties of the distal tibia and tibial shaft of both legs were assessed by pQCT (XCT‐2000; Stratec Medizintechnik, Pforzheim, Germany). The pQCT device was calibrated daily using a standard phantom and monthly using a cone phantom provided by the manufacturer. The distal tibia was defined as 5% and tibial shaft as 55% of the measured tibial length proximal to the distal end plate. The scan line was adjusted using the scout view of the pQCT system. Tibial length was defined as the distance between the lateral malleolus and the lateral condyle of the tibia. A single (2.4‐mm) axial slice with a voxel size of 0.8 × 0.8 mm, typical tube voltage of 46 kV, tube current of 0.3 mA, and scan speed of 20 mm/s was obtained.

The images were analyzed with an automated threshold‐free cortical bone detection method (the outer boundary detection and subsequent shrinking [OBS] procedure, OBS cortical bone detection 2.1).^27^, ^28^ For the distal tibia, compressive bone strength index (BSI, g^2^/cm^4^ = vBMD_TOT_^2^ × CSA_TOT_),^29^, ^30^ total volumetric BMD (vBMD_TOT_, mg/cm^3^), and total cross‐sectional area (CSA_TOT_, mm^2^) were determined. The parameters for the tibia midshaft site were the strength‐strain index (SSI, mm^3^; density‐weighted polar section modulus), reflecting the bone's resistance to bending and torsional loads, cortical vBMD (vBMD_CO_), and the ratio of cortical to total area (CSA_CO_/CSA_TOT)_. The root mean square coefficient of variation (CV_RMS_) for the BMD, structure, and strength index measurements in our laboratory ranges from 0.4% to 1.6%.^31^

### Health, fracture status, and anthropometry

At baseline, during a medical examination performed by a research nurse and a physician, the presence of chronic conditions, use of prescription medications, fracture date and status, and type and date of surgery were confirmed with a prestructured questionnaire, current prescriptions, and medical records. Contraindications for the physical performance assessments and the intervention were evaluated according to the American College of Sports Medicine guidelines.^32^ Blood count, C‐reactive protein, and hemoglobin analyses were performed to evaluate possible acute conditions before the performance measurements. Body height and weight were measured using a stadiometer and a digital scale, and BMI was calculated as body weight divided by height squared (kg/m^2^). Body fat percentage was assessed with a bioimpedance devise with eight polar electrodes (BC‐418; TANITA, Tokyo, Japan). Blood samples were drawn from the antecubital vein in the morning. Specimens were centrifuged and frozen at −80°C until analysis. Serum concentrations of 25‐hydroxyvitamin D (25OHD; nmol/L) and parathyroid hormone (PTH, ng/L) were determined at baseline using an electrochemiluminescence immunoassay and a chemiluminescence immunoassay (Modular Analytics E170; Roche Diagnostics, Mannheim, Germany), respectively. The intra‐assay CV for 25OHD was 1.1% to 2.0% (26.7 to 261 ng/L), and for PTH it was 2.2% to 6.8% (16.9 to 168 nmol/L). Smoking history and alcohol consumption were assessed by questionnaire.

### Physical activity and physical performance

Current level of physical activity (PA) was assessed by a slightly modified Grimby scale^33^ with seven response alternatives: (1) mainly resting, (2) most activities performed in a sitting position, (3) light PA twice a week at most, (4) moderate PA or housework about 3 hours a week, (5) moderate PA or housework at least 4 hours a week or heavy PA ≤ 4 hours a week, (6) physical exercise or heavy leisure time PA several times a week, and (7) competitive sports several times a week. The responses were recoded for analyses as inactivity (categories 1 to 2), light PA (category 3), and moderate‐to‐heavy PA (categories 4 to 7).^34^ Physical performance was assessed according to the Short Physical Performance Battery, which includes habitual walking speed, chair rise, and balance tests.^35^ A higher score (range, 0 to 12) indicates better performance. Maximal isometric knee extension force of the fractured and nonfractured leg was measured in a sitting position using an adjustable dynamometer chair (Good Strength; Metitur Ltd, Palokka, Finland).^36^ The ankle was attached to a strain‐gauge system with the knee angle fixed at 120 degrees. After two to three submaximal practice trials, three maximal trials were recorded and further trials performed until no further improvement occurred. Each maximal effort was maintained for 2 to 3 s, separated by a 30‐s rest. The highest recorded force value was used for the analysis. Leg extension power of each leg was measured with the Nottingham Leg Extensor Power Rig in an upright sitting position.^37^, ^38^ The distance between the seat and the push‐pedal was adjusted for leg length. The measurement was repeated until no further improvement occurred; the best performance was used in the analyses. In our laboratory, the test–retest CVs for the force and power measurements were 6%^36^ and 8%,^38^ respectively.

### Intervention

The intervention group received a year‐long, physical rehabilitation program aimed at restoring mobility and physical functional capacity to the pre hip fracture level.^25^, ^26^ The individually tailored program comprised an evaluation and modification of environmental hazards,^39^ guidance for safe walking, nonpharmacological pain management, motivational physical activity counseling, and a progressive home exercise program. The intervention took place in the participants’ homes and included five to six home visits by a physiotherapist.

The progressive home exercise program comprised strengthening and stretching exercises for the lower limb muscles, balance training in the standing position, and functional exercises including walking, reaching, turning in different directions, and stair climbing. All exercises were weight‐bearing. The program was progressively increased in intensity and demandingness 4 to 5 times. The strengthening and stretching exercises (performed on the same day, 3 times per week), and the balance and functional exercises (performed on the same day, 2 to 3 times per week) were performed on nonconsecutive days. Each training session lasted approximately 30 minutes. The strengthening exercises included knee extension and flexion, hip abduction, plantar flexion, chair rising, and squatting. In the strengthening exercises, the resistance was progressively increased with resistance bands of three different strengths. Functional exercises were performed only during the first 12 weeks. All participants in the intervention group kept a daily exercise diary. Motivational physical activity counseling was delivered as two face‐to‐face sessions (at 3 and 6 months) and three phone contacts (at 4, 8, and 10 months).

### Control condition

Information on standard care after hip fracture was collected by interview at baseline. In total, 68% of the intervention group and 71% of the controls (*p *= 0.813) reported having received a home exercise program from the hospital or the health care center. Typically, the program comprised five to seven exercises for the lower limbs (mostly the fractured leg) without additional resistance or progression.^26^ Compliance with the home exercise program was not monitored and the program was not increased in intensity. Five intervention participants and seven controls were referred for physiotherapy.

### Statistical analysis

The study power, calculated for the main outcome, mobility limitation, was 78%. Mean values, standard deviations and standard errors were calculated using standard procedures. All outcome variables were analyzed according to the intention‐to‐treat principles. Baseline characteristics were compared by cross‐tabulation and chi‐square tests for discrete variables, by independent samples *t* test for normally distributed data, and by the Mann‐Whitney *U* test for non‐normally distributed continuous data. The normality of the distributions was tested with the Shapiro‐Wilk test. The effect of the intervention was assessed using an interaction term (group × time) in a general linear model for longitudinal data estimated in Mplus, version 7.4.^40^ The models were adjusted by age, keeping the age effect constant over time. An additional analysis was performed by adjusting the models by age, sex, and body weight, but the results were not different from the main analysis (data not shown). We assumed that missing data were missing‐not‐at‐random (MNAR); hence, for example, we used the maximum likelihood‐based pattern‐matching model^41^ to include the data from dropouts in the statistical data analysis up to the time of loss to follow‐up. The main reasons for missing bone data were inability to perform the measurements, inaccurate positioning of the leg, a technically invalid pQCT scan, substantial movement artifacts, and metal in tissues in the scanned region. For the distal tibia, 154 valid scans were obtained at baseline, 133 at 3 months, 137 at 6 months, and 130 at 12 months. For the midshaft site, the corresponding numbers were 156, 136, 134, and 130. A per protocol analysis on the effect of the intervention was also performed. For this analysis, only subjects whose overall compliance with physical exercises was over 70% (*n *= 16) were chosen from the intervention group. In addition, sensitivity analyses were performed by restricting the analyses to women (intervention group, *n *= 31; control group, *n* = 32). Descriptive analyses were performed using SPSS 24.0 software (IBM, Armonk, NY, USA) and the general linear model extended for MNAR longitudinal data was analyzed using Mplus 7.4 with the significance level set to 5%. Mean changes were calculated as (follow‐up − baseline), and mean percentage changes were calculated as [(follow‐up − baseline)/baseline × 100]. Side‐to‐side differences in bone variables were defined as (nonfractured leg − fractured leg). Compliance with the intervention was calculated using the following formula: (number of performed exercises)/(expected number of exercises) × 100.

## Results

No differences were observed between the intervention and control groups in baseline characteristics (Table 1). Mean serum concentrations of 25OHD and PTH were normal. In total, 28 participants had a serum 25OHD level below 50 nmol/L. Seven of these 28 had values below 25 nmol/l. Based on medical records and questionnaires collected at baseline and at 3, 6, and 12 months, 13 intervention participants and 14 controls reported taking bisphosphonates during the 12‐month intervention. In addition, one participant in the intervention group reported receiving strontium ranelate. In the per protocol analysis, no significant between‐group differences were observed in baseline characteristics.

**Table 1:** Baseline characteristics of the participants

	Intervention (*n* = 40)	Control (*n* = 41)
Age, years	80.9 (7.7)	79.1 (6.4)
Women, *n* (%)	31 (78)	32 (78)
Height, cm	160.9 (8.9)	160.3 (9.1)^a^
Weight, kg	65.8 (11.9)	65.9 (11.3)
BMI, kg/m^2^	25.3 (3.6)	25.6 (3.9)^a^
Body fat, %	30.5 (7.1)^b^	32.2 (5.8)^b^
Hemoglobin, g/L	127 (13)	130 (13)
Lowest hemoglobin after surgery, g/L	98 (11)	99 (15)
Smoking, *n* (%)		
Never	34 (85)	30 (73)
Former	4 (10)	6 (15)
Current	2 (5)	5 (12)
Number of chronic diseases	3 (2)	3 (2)
Current bisphosphonate use, *n* (%)	9 (23)	7 (17)
Oral corticosteroid use, *n* (%)	1 (2.5)	1 (2.4)
Serum‐25OHD, nmol/L	57 (22)^c^	54 (24)^d^
Serum‐PTH, ng/L	49 (23)^c^	49 (23)^d^
Site of fracture, *n* (%)		
Femoral neck	27 (68)	25 (61)
Pertrochanteric	13 (33)	16 (39)
Type of surgery, *n* (%)		
Internal fixation	19 (48)	19 (46)
Hemiarthroplasty	15 (38)	18 (44)
Total arthroplasty	6 (15)	4 (10)
Time since fracture (days)	68 (16)	71 (37)
Level of physical activity, *n* (%)		
Inactivity (mostly sitting)	15 (38)	11 (28)
Light activity	23 (58)	25 (63)
Moderate‐to‐heavy activity	2 (5)	4 (10)
Physical performance		
SPPB score	5.8 (2.5)	6.6 (2.2)
Knee extension force, *N*		
Fractured side	185.1 (73.1)^e^	168.3 (71.6)^a^
Nonfractured side	240.4 (93.4)^b^	228.3 (83.9)^a^
Leg extension power, W		
Fractured side	55.9 (29.5)^c^	51.1 (28.6)^b^
Nonfractured side	73.9 (37.1)^d^	73.8 (40.6)^f^

Values are means (SD) or n (%) unless otherwise noted.

^a^ *n* = 40.

^b^ *n* = 38.

^c^ *n* = 32.

^d^ *n* = 36.

^e^ *n* = 34.

^f^ *n* = 39.

SPPB = short physical performance battery.

### Intervention adherence and adverse events

During the 12‐month study, one intervention participant and two controls dropped out for personal reasons, and one intervention participant died from cardiac failure unrelated to the intervention before the 12‐month measurements. No intervention‐related adverse events occurred. Four intervention participants were suspended by a physician for medical reasons during the first 6 months of the study. Two of them returned to the intervention (revision operation, femoral fracture), but 2 were unable to continue (pneumonia and new hip fracture, pulmonary embolism). During the final 6 months, 5 participants were suspended (pubic bone fracture, urinary tract infection, cerebral infarction, cardiac failure, sacrum strain fracture) and none of them returned. In the control group, four revision operations were performed.

### Compliance with physical exercises

Overall compliance with the exercises was 50% for the strengthening, 45% for the stretching, 54% for the balance, and 69% for the functional exercises. During the first 6 months, compliance was 61% for the strengthening, 53% for the stretching, and 65% for the balance exercises. During the last 6 months, the corresponding values were 39%, 37%, and 43%. Compliance with the first face‐to‐face physical activity counseling session was 97%, and with the following sessions as follows: 90% (first phone contact), 87% (second face‐to‐face), and 85% (second phone contact), and 79% (third phone contact).

### Muscle force and power

The intervention had no effect (group × time) on maximal isometric knee extension force or leg extension power. Fractured side force and power increased significantly in both groups (time effect, 12 months, *p* < 0.001): 24% and 32%, respectively, in the intervention group and 26% and 35% in the control group. Leg extension power of the nonfractured leg increased significantly in both groups (time effect, 12 months, *p *= 0.001): 4% in the intervention group and 15% in the control group.

### Bone properties

**Figure 2:**
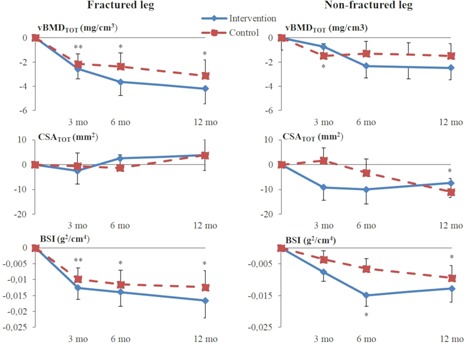
Mean change relative to baseline values for vBMD_TOT_, CSA_TOT_, and compressive bone strength index of the distal tibia. (Mean, SE) *p < 0.05, **p < 0.01, ***p < 0.001 for the time effect at different time points. Intention‐to‐treat analysis. vBMD_TOT_ = total volumetric BMD; CSA_TOT_ = total cross‐sectional area; BSI = compressive bone strength index.

The intervention had no effect (group × time) on the distal tibia or midtibial bone traits (Tables 2 and 3). At the distal site (Table 2 and Fig. 2) at 3 months, vBMD_TOT_ of both legs and BSI of the fractured leg had decreased significantly in both groups, whereas at 6 months, vBMD_TOT_ of the fractured leg and BSI of both legs had decreased in both groups. At 12 months, vBMD_TOT_ of the fractured leg, CSA_TOT_ of the nonfractured leg and BSI of both legs had decreased significantly in both groups. The mean decrease in vBMD_TOT_ from baseline to 12 months on the fractured side was 1.9% in the intervention group and 1.5% in the control group. The values for CSA_TOT_ on the nonfractured side were 0.7% and 1.0%. In the intervention group, the mean decrease in BSI was 3.1% in the fractured leg and 2.3% in the nonfractured leg, whereas in the control group the corresponding values were 2.7% and 2.0%. A significant group difference over follow‐up time was observed in side‐to‐side difference in CSA_TOT_ favoring the nonfractured leg in the intervention group and fractured leg in the control group, respectively.

**Table 2:** Distal tibia bone traits at baseline and at different follow‐up points, and *p*‐values for group, time and interaction effects. Intention‐totreat analysis

			vBMD_TOT (_mg/cm^3^)			CSA_TOT_ (mm^2^)			BSI (g^2^/cm^4^)		
Group	Time		Fractured leg	Nonfractured leg	Side‐to‐side difference	Fractured leg	Nonfractured leg	Side‐to‐side difference	Fractured leg	Nonfractured leg	Side‐to‐side difference
Intervention	Baseline		225 (8)	227 (8)	0.6 (2.4)	1020 (26)	1046 (26)	25 (8)	0.54 (0.04)	0.56 (0.04)	0.011 (0.010)
	3 months		222 (8)	226 (8)	2.5 (2.4)	1017 (26)	1037 (27)	16 (8)	0.53 (0.04)	0.55 (0.04)	0.017 (0.010)
	6 months		221 (8)	224 (8)	1.8 (2.3)	1022 (25)	1036 (25)	10 (7)	0.53 (0.04)	0.54 (0.04)	0.010 (0.010)
	12 months		221 (8)	224 (8)	3.0 (2.3)	1023 (25)	1039 (25)	9 (9)	0.53 (0.04)	0.54 (0.04)	0.017 (0.010)
Control	Baseline		207 (8)	209 (8)	5.1 (2.3)	1032 (26)	1033 (25)	−5 (8)	0.46 (0.04)	0.47 (0.04)	0.019 (0.010)
	3 months		205 (8)	207 (8)	5.7 (2.3)	1032 (26)	1035 (26)	−2 (8)	0.45 (0.04)	0.47 (0.04)	0.025 (0.010)
	6 months		204 (8)	208 (8)	5.7 (2.3)	1031 (25)	1030 (24)	−4 (7)	0.45 (0.04)	0.47 (0.04)	0.022 (0.010)
	12 months		204 (8)	207 (8)	6.6 (2.2)	1036 (25)	1022 (24)	−18 (8)	0.45 (0.04)	0.46 (0.04)	0.020 (0.009)
*p*‐value	Group		0.109	0.119	0.178	0.733	0.718	0.006	0.131	0.123	0.570
	Time	3	0.007	0.023	0.527	0.914	0.718	0.623	0.005	0.183	0.127
		6	0.033	0.083	0.573	0.797	0.735	0.910	0.009	0.043	0.502
		12	0.012	0.176	0.127	0.541	0.043	0.079	0.016	0.018	0.949
	Group × time	3	0.714	0.432	0.320	0.785	0.132	0.184	0.579	0.312	0.974
		6	0.424	0.347	0.688	0.604	0.404	0.096	0.700	0.076	0.518
		12	0.553	0.540	0.544	0.999	0.659	0.813	0.568	0.567	0.348

Values are estimated mean (SE). Side‐to‐side differences calculated as (nonfractured leg – fractured leg).

vBMD_TOT_ = total volumetric BMD; CSA_TOT_ = total cross‐sectional area; BSI = compressive bone strength index.

**Table 3:** Tibial mid‐shaft bone traits at baseline and at different follow‐up points, and p‐values for group, time and interaction effects. Intentionto‐ treat analysis

			vBMD_CO_ (mg/cm^3^)			CSA_CO_/CSA_TOT_			SSI (mm^3^)		
Group	Time		Fractured leg	Nonfractured leg	Side‐to‐side difference	Fractured leg	Nonfractured leg	Side‐to‐side difference	Fractured leg	Nonfractured leg	Side‐to‐side difference
Intervention	Baseline		1050 (10)	1057 (12)	5.7 (6)	0.576 (0.015)	0.581 (0.016)	0.007 (0.008)	1524 (70)	1571 (72)	43 (27)
	3 months		1047 (11)	1053 (12)	4.7 (6)	0.574 (0.016)	0.582 (0.015)	0.010 (0.008)	1513 (73)	1562 (74)	50 (26)
	6 months		1043 (11)	1051 (12)	5.5 (7)	0.570 (0.016)	0.578 (0.016)	0.011 (0.008)	1501 (72)	1558 (74)	58 (26)
	12 months		1039 (12)	1049 (12)	7.5 (7)	0.565 (0.016)	0.575 (0.016)	0.012 (0.008)	1497 (73)	1549 (73)	48 (26)
Control	Baseline		1035 (11)	1032 (11)	‐1.7 (6)	0.552 (0.015)	0.551 (0.015)	0.005 (0.007)	1456 (71)	1460 (69)	11 (26)
	3 months		1029 (11)	1030 (12)	1.1 (6)	0.550 (0.016)	0.551 (0.015)	0.007 (0.008)	1458 (73)	1463 (72)	15 (25)
	6 months		1026 (11)	1030 (12)	3.6 (6)	0.552 (0.016)	0.547 (0.015)	0.003 (0.007)	1446 (72)	1456 (72)	16 (25)
	12 months		1020 (12)	1027 (12)	9.0 (7)	0.546 (0.016)	0.544 (0.015)	0.003 (0.008)	1429 (73)	1441 (70)	21 (25)
*p*‐value	Group		0.306	0.129	0.403	0.278	0.165	0.829	0.500	0.268	0.397
	Time	3	0.028	0.444	0.502	0.153	0.883	0.317	0.800	0.679	0.749
		6	0.004	0.475	0.108	0.688	0.085	0.386	0.324	0.656	0.644
		12	<0.001	0.099	0.004	0.025	0.001	0.547	0.012	0.021	0.273
	Group × time	3	0.531	0.671	0.530	0.622	0.624	0.969	0.324	0.318	0.864
		6	0.829	0.328	0.257	0.069	0.778	0.105	0.153	0.496	0.516
		12	0.482	0.475	0.108	0.215	0.833	0.144	0.969	0.864	0.685

Values are estimated mean (SE). Side‐to‐side differences calculated as (non‐fractured leg – fractured leg).

vBMD_CO_ = cortical volumetric BMD, CSA_CO_/CSA_TOT_ = ratio of cortical to total area, SSI = strength‐strain index.

**Figure 3:**
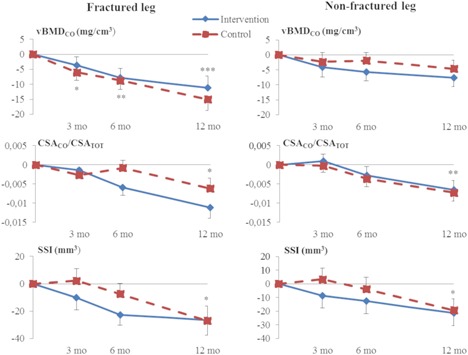
Mean change relative to baseline values for vBMD_CO_, CSA_CO_/CSA_TOT_, and strength‐strain index of the tibial midshaft. (Mean, SE) *p < 0.05, **p < 0.01, ***p < 0.001 for the time effect at different time points. Intention‐to‐treat analysis. vBMD_TOT_ = total volumetric BMD; CSA_CO_/CSA_TOT_ = ratio of cortical to total area; SSI = strength–strain index.

At the midshaft site (Table 3 and Fig. 3), vBMD_CO_ of the fractured side leg decreased significantly over time in both groups at 3, 6, and 12 months, whereas CSA_CO_/CSA_TOT_ and SSI of both legs decreased significantly over 12 months in both groups. The mean decrease in vBMD_CO_ from baseline to 12 months on the fractured side was 1.1% in the intervention group and 1.5% in the control group. On the fractured side, the corresponding values for CSA_CO_/CSA_TOT_ were 1.9% and 1.1%, and on the nonfractured side 1.1% and 1.3%, for the intervention group and controls, respectively. SSI on the fractured side decreased by 1.7% in the intervention group and 1.9% in the control group, whereas on the nonfractured side the decrease was 1.4% and 1.3%. Side‐to‐side difference in vBMD_CO_ increased significantly over 12 months in both groups favoring the nonfractured leg.

The changes in the bone outcomes were not systematically associated with the changes in maximal isometric knee extension force and leg extension power (data not shown).

No significant interaction effects were observed in the analyses restricted to women only, except for CSA_TOT_ of the nonfractured leg and CSA_CO_/CSA_TOT_ of the fractured leg. In the intervention group compared with controls, CSA_TOT_ of the nonfractured leg decreased significantly at 3 months (*p *= 0.047), whereas at 6 months CSA_CO_/CSA_TOT_ of the fractured leg decreased significantly more in the intervention group compared with controls (*p *= 0.019).

### Per protocol analysis

No intervention effect was observed in the distal tibia or midtibial bone traits (Supplemental Tables S1 and S2).

## Discussion

This 12‐month home‐based physical rehabilitation program on mobility recovery had no effect on the distal tibia or tibial midshaft bone traits of community‐dwelling men and women over age 60 years recovering from a hip fracture. The bone structural and densitometric traits of both legs continued to deteriorate during the year following the fracture. At both bone sites, bone loss was more evident in the fractured leg, especially in total and cortical density.

The present findings are in line with those previously reported for the effects of physical rehabilitation on bone traits after hip fracture. To our knowledge, only two intervention studies have been conducted.^15^, ^16^ Orwig and colleagues^16^ conducted an RCT of a 12‐month low‐intensity home exercise program for older women with a recent hip fracture. Compared with controls receiving usual care, the intervention did not result in significant changes in contralateral hip aBMD. Similarly, a more‐intensive 6‐month outpatient rehabilitation program including progressive resistance training did not improve hip or total body aBMD compared with a low‐intensity home‐exercise program.^15^ Studies with osteoporotic participants comparable to hip fracture patients have also revealed minor or no effects on bone density.^12^, ^42^ A nine‐month program including progressive strength and endurance training did not increase aBMD in fragile, elderly men and women.^13^ Similarly, a long‐term (2.5 years) impact training program had no effect on aBMD in older women with weak bones, although the BMC of the femoral neck decreased significantly less in the intervention group compared with controls.^14^

The absence of training‐induced improvements in previous studies as well as in the present study could be related to the low muscular capacity of the elderly subjects, which may have limited their ability to produce the peak forces required for bone adaptation. In addition, the programs may have lacked intensity and specificity for bone adaptation. In contrast, our previous study on middle‐aged and older male athletes with above‐average muscle characteristics^21^ showed significant improvements in tibial structure and strength after novel, intensive strength and sprint training, suggesting that in the presence of high‐intensity loading and with sufficient muscle strength the adaptability of aging bone structure is maintained. This potential explanation is also supported by animal and human studies demonstrating that given the right stimulus, bone mechanoresponsiveness remains largely unaltered with aging,^43^, ^44^ although some decrease in mechanosensitivity may occur.^45^ In the present study, the primary target was not bone traits per se, the intensity of the rehabilitation program was relatively low, and no effects on bone structure and strength were observed. Although all the exercises were weight‐bearing and elastic resistance bands of different strengths were used, it is plausible that the program did not provide a sufficient stimulus for osteogenic adaptations. Although muscle force and power increased significantly over time in both groups (no between‐group differences), the levels might nevertheless have been too low and the strains generated not novel enough to stimulate bone formation. Moreover, compliance with the strengthening as well as other physical exercises may not have been sufficient for bone adaptation, especially during the last 6 months of the intervention.

In this study, as in our previous cross‐sectional study on hip fracture patients,^2^ bone deterioration was more evident in the fractured than nonfractured leg, both at the distal tibia and midshaft sites. At the midshaft site, a side‐to‐side difference in cortical density increased significantly over time in both groups favoring the nonfractured leg. In our previous cross‐sectional study,^2^ lower bone characteristics were manifested as decreased BMC and geometrical properties, and no side‐to‐side or between‐group differences were observed in vBMD. In the present study, by contrast, vBMD on the fractured side decreased significantly at both bone sites. At the midshaft site, bone deterioration in the nonfractured leg was manifested as a decrease in the SSI and the ratio of cortical to total area, whereas on the fractured side, bone loss was also evident in volumetric cortical density. Based on the results of high‐resolution CT exploration of age‐related bone loss, which have shown intracortical bone loss and resulting increased cortical porosity,^46^ we assumed that in our sample intracortical bone loss was more pronounced in the fractured leg than nonfractured leg.

Several issues merit further discussion. Our study sample was rather heterogeneous in participant age, physical functional capacity, and bone properties, factors that help to explain the large individual variability in the bone results. The inclusion of both sexes also increased variability and may have affected the results. Owing to their larger skeletal size and higher bone mass, men generally have more robust bone characteristics. In addition, the changes in bone density and structure after hip fracture may in part be different between the sexes.^4^, ^5^ The differences in posthip fracture BMD changes could also be related to the accelerated bone loss in older men compared with the attenuated decline in women for whom bone loss follows menopause and thus occurs earlier.^4^ Furthermore, one‐third of our participants were using bisphosphonates (no difference between the groups), which again may have affected the results. Bisphosphonates increase BMD by inhibiting bone resorption by osteoclasts, which may suppress bone remodeling and, after long‐term usage, possibly limit the bone cell response to exercise. In the present study, the results restricted to women did not differ from the main analysis. The sample size, especially in the restricted analyses was, however, rather small. The number of participants was insufficient for subsample analysis of the effect of bisphosphonates on the results.

This study has its limitations. Most importantly, the study reports secondary outcomes of an RCT. The home exercise program was not specifically designed to improve bone strength, and probably lacked the intensity and specificity needed for bone adaptation. Furthermore, owing to the inclusion criteria, our participants were probably healthier than hip fracture patients on average, a factor that should be considered when generalizing the results. However, for the frailest patients, a program of this kind would not be advisable. Inclusion of measures of the proximal femur would have added value to our study. Because of the imaging modality used, our results are not fully comparable with those of previous studies. A few methodological considerations also be kept in mind when interpreting the results. pQCT, the imaging method used in this study, is susceptible to partial volume effect and beam hardening. In addition, a higher scan resolution would have provided more detailed results. Finally, the amount of missing data was considerable, partly because of the frailty of the study population. We were, however, able to account for this by using a specifically tailored maximum likelihood estimation method.

The strengths of this study include a randomized controlled study design and the use of a 3D imaging modality to examine changes in bone geometry and volumetric density. Our study was the first trial to examine the effect of physical exercise on bone properties of both the fractured and nonfractured leg in hip fracture patients. Furthermore, we used a theory‐based approach to the assessments and the intervention, and investigated a topic that has high clinical and societal relevance. Moreover, the home‐based physical rehabilitation program eliminated the burden of traveling to a facility, it was individually tailored, and it included visits by a physiotherapist as well as motivational physical activity counseling. Despite no effect on bone, the rehabilitation program increased physical activity^34^ and improved mobility recovery.^25^ The intervention was well‐tolerated, the program was feasible in the home setting, and the dropout rate was low. Compliance with the home exercises was moderate and comparable to that reported in other similar studies.^47^, ^48^ Compliance with the physical activity counseling was excellent. Finally, the one‐year follow‐up was of sufficient duration to detect changes in bone, and bone data were gathered at multiple time points.

In conclusion, our home‐based physical rehabilitation was unable to prevent bone deterioration in older people after hip fracture. Tibial bone traits, both cortical and trabecular, continued to weaken during the year following the fracture, on both the fractured and nonfractured side. Together with decreased muscle strength, deterioration in bone properties markedly increases the risk for a second fracture; hence, specific interventions targeting bones and muscles should be developed to maximize postfracture recovery and minimize deterioration. Improving muscle function and balance to reduce the risk of recurrent falls and fractures may be a more feasible intervention target after hip fracture, especially because preventing bone deterioration seems unlikely. More research is, however, needed to find out whether fragile bones, such as those in older hip fracture patients, are able to adapt to increased physical loading, and what type of exercise would be safe, feasible, and effective.

## Disclosures

All authors state that they have no conflicts of interest.

## Supporting information

Supporting Table S1.

Supporting Table S2.
