# Supplementary material for: Effects of a Home‐Based Physical Rehabilitation Program on Tibial Bone Structure, Density, and Strength After Hip Fracture: A Secondary Analysis of a Randomized Controlled Trial
Source: JBMR Plus. 2019 Mar 6;3(6):e10175. doi: 10.1002/jbm4.10175 (PMC6636770; doi:10.1002/jbm4.10175)
Supplement: Supplementary file 1 — Supporting Table S1. [file JBM4-3-na-s001.docx]

**Supplemental Table 1.** Distal tibia bone traits at baseline and at different follow-up points, and p-values for group, time and interaction effects. Per protocol analysis.

|  |  | | **vBMD_TOT (_mg/cm^3^)** | | | **CSA_TOT_ (mm^2^)** | | | **BSI (g^2^/cm^4^)** | | |
| --- | --- | --- | --- | --- | --- | --- | --- | --- | --- | --- | --- |
| Group | Time | | Fractured leg | Non-fractured leg | Side-to-side difference | Fractured leg | Non-fractured leg | Side-to-side difference | Fractured leg | Non-fractured leg | Side-to-side difference |
| Intervention | Baseline | | 218 (12) | 220 (13) | 1.6 (4.1) | 1030 (42) | 1043 (44) | 26 (13) | 0.52 (0.06) | 0.53 (0.04) | 0.016 (0.017) |
|  | 3 months | | 218 (12) | 219 (13) | 1.9 (4.0) | 1028 (43) | 1042 (46) | 23 (14) | 0.52 (0.06) | 0.52 (0.04) | 0.016 (0.017) |
|  | 6 months | | 217 (12) | 219 (13) | 2.0 (3.9) | 1032 (41) | 1027 (42) | 6 (12) | 0.51 (0.04) | 0.51 (0.04) | 0.007 (0.017) |
|  | 12 months | | 218 (12) | 220 (13) | 3.3 (3.9) | 1034 (39) | 1036 (42) | 10 (14) | 0.52 (0.04) | 0.53 (0.04) | 0.018 (0.016) |
| Control | Baseline | | 207 (8) | 210 (8) | 5.1 (2.5) | 1029 (27) | 1031 (26) | -5 (8) | 0.46 (0.04) | 0.48 (0.06) | 0.020 (0.010) |
|  | 3 months | | 205 (8) | 208 (8) | 5.7 (2.4) | 1028 (27) | 1033 (27) | -2 (9) | 0.45 (0.04) | 0.47 (0.06) | 0.025 (0.010) |
|  | 6 months | | 205 (8) | 208 (8) | 5.7 (2.4) | 1028 (26) | 1027 (26) | -4 (8) | 0.45 (0.04) | 0.47 (0.06) | 0.023 (0.010) |
|  | 12 months | | 204 (8) | 208 (8) | 6.6 (2.4) | 1033 (25) | 1019 (25) | -19 (9) | 0.45 (0.04) | 0.47 (0.06) | 0.019 (0.010) |
| *p*-value | Group | | 0.436 | 0.499 | 0.462 | 0.928 | 0.803 | 0.048 | 0.453 | 0.474 | 0.858 |
|  | Time | 3 | 0.005 | 0.020 | 0.511 | 0.902 | 0.657 | 0.609 | 0.002 | 0.124 | 0.124 |
|  |  | 6 | 0.023 | 0.074 | 0.526 | 0.783 | 0.540 | 0.888 | 0.005 | 0.026 | 0.465 |
|  |  | 12 | 0.010 | 0.140 | 0.095 | 0.527 | 0.030 | 0.058 | 0.010 | 0.006 | 0.940 |
|  | Group × time | 3 | 0.469 | 0.444 | 0.859 | 0.923 | 0.701 | 0.623 | 0.128 | 0.973 | 0.411 |
|  |  | 6 | 0.227 | 0.890 | 0.902 | 0.987 | 0.247 | 0.081 | 0.216 | 0.253 | 0.157 |
|  |  | 12 | 0.606 | 0.425 | 0.907 | 0.451 | 0.678 | 0.894 | 0.145 | 0.214 | 0.752 |

Values are estimated mean (SE). vBMD_TOT_ = total volumetric BMD, CSA_TOT_ = total cross-sectional area, BSI = compressive bone strength index. Side-to-side differences calculated as (non-fractured leg – fractured leg).
