# Supplementary material for: Effects of a Home‐Based Physical Rehabilitation Program on Tibial Bone Structure, Density, and Strength After Hip Fracture: A Secondary Analysis of a Randomized Controlled Trial
Source: JBMR Plus. 2019 Mar 6;3(6):e10175. doi: 10.1002/jbm4.10175 (PMC6636770; doi:10.1002/jbm4.10175)
Supplement: Supplementary file 2 — Supporting Table S2. [file JBM4-3-na-s002.docx]

**Supplemental Table 2.** Tibial mid-shaft bone traits at baseline and at different follow-up points, and p-values for group, time and interaction effects. Per protocol analysis.

|  |  | | **vBMD_CO_ (mg/cm^3^)** | | | **CSA_CO_/CSA_TOT_** | | | **SSI (mm^3^)** | | |
| --- | --- | --- | --- | --- | --- | --- | --- | --- | --- | --- | --- |
| Group | Time | | Fractured leg | Non-fractured leg | Side-to-side difference | Fractured leg | Non-fractured leg | Side-to-side difference | Fractured leg | Non-fractured leg | Side-to-side difference |
| Intervention | Baseline | | 1048 (18) | 1048 (21) | -0.8 (10.6) | 0.577 (0.026) | 0.588 (0.027) | 0.014 (0.013) | 1493 (117) | 1555 (117) | 50 (45) |
|  | 3 months | | 1047 (18) | 1042 (21) | -3.4 (10.6) | 0.574 (0.026) | 0.588 (0.026) | 0.018 (0.012) | 1491 (122) | 1544 (121) | 52 (40) |
|  | 6 months | | 1044 (19) | 1040 (21) | -2.5 (10.8) | 0.573 (0.026) | 0.583 (0.027) | 0.015 (0.012) | 1481 (120) | 1550 (122) | 61 (43) |
|  | 12 months | | 1042 (20) | 1045 (21) | 1.4 (11.1) | 0.570 (0.026) | 0.581 (0.026) | 0.014 (0.013) | 1479 (121) | 1533 (118) | 44 (43) |
| Control | Baseline | | 1038 (11) | 1036 (12) | -1.6 (6.5) | 0.556 (0.016) | 0.556 (0.016) | 0.005 (0.008) | 1457 (74) | 1461 (71) | 11 (28) |
|  | 3 months | | 1032 (12) | 1033 (13) | 0.9 (6.6) | 0.553 (0.017) | 0.556 (0.016) | 0.007 (0.008) | 1459 (77) | 1464 (73) | 15 (25) |
|  | 6 months | | 1029 (12) | 1033 (13) | 3.6 (6.6) | 0.555 (0.017) | 0.553 (0.016) | 0.003 (0.008) | 1451 (76) | 1457 (74) | 15 (26) |
|  | 12 months | | 1023 (12) | 1031 (13) | 9.0 (6.8) | 0.550 (0.017) | 0.549 (0.016) | 0.003 (0.008) | 1431 (77) | 1442 (72) | 21 (27) |
| *p*-value | Group | | 0.608 | 0.605 | 0.945 | 0.483 | 0.306 | 0.548 | 0.796 | 0.493 | 0.466 |
|  | Time | 3 | 0.027 | 0.429 | 0.552 | 0.149 | 0.895 | 0.324 | 0.826 | 0.736 | 0.732 |
|  |  | 6 | 0.006 | 0.473 | 0.074 | 0.691 | 0.096 | 0.385 | 0.304 | 0.613 | 0.672 |
|  |  | 12 | <0.001 | 0.083 | 0.004 | 0.013 | 0.008 | 0.476 | 0.011 | 0.010 | 0.262 |
|  | Group × time | 3 | 0.396 | 0.602 | 0.525 | 0.947 | 0.868 | 0.663 | 0.775 | 0.405 | 0.943 |
|  |  | 6 | 0.378 | 0.304 | 0.207 | 0.322 | 0.822 | 0.491 | 0.645 | 0.987 | 0.715 |
|  |  | 12 | 0.220 | 0.711 | 0.237 | 0.849 | 0.896 | 0.620 | 0.513 | 0.838 | 0.374 |

Values are estimated mean (SE). vBMD_CO_ = cortical volumetric BMD, CSA_CO_/CSA_TOT_ = ratio of cortical to total area, SSI = strength-strain index. Side-to-side differences calculated as (non-fractured leg – fractured leg).
